# Extensive Mammalian Ancestry of Pandemic (H1N1) 2009 Virus

**DOI:** 10.3201/eid1602.091141

**Published:** 2010-02

**Authors:** Natalia A. Ilyushina, Jeong-Ki Kim, Nicholas J. Negovetich, Young-Ki Choi, Victoria Lang, Nicolai V. Bovin, Heather L. Forrest, Min-Suk Song, Philippe Noriel Q. Pascua, Chul-Joong Kim, Robert G. Webster, Richard J. Webby

**Affiliations:** St. Jude Children’s Research Hospital, Memphis, Tennessee, USA (N.A. Ilyushina, J.-K. Kim, N.J. Negovetich, V. Lang, H.L. Forrest, R.G. Webster, R.J. Webby); D.I. Ivanovsky Institute of Virology, Moscow, Russia (N.A. Ilyushina); Korea Research Institute of Bioscience and Biotechnology, Daejeon, Republic of Korea (J.-K. Kim); Chungbuk National University College of Medicine and Medical Research Institute, Cheongju, Republic of Korea (Y.-K. Choi, M-S Song, P.N.Q. Pascua); Shemyakin Institute of Bioorganic Chemistry, Moscow (N.V. Bovin); Chungnam National University College of Veterinary Medicine, Daejeon (C.-J. Kim); University of Tennessee Health Science Center, Memphis (R.G. Webster, R.J. Webby); 1 These authors contributed equally to this article.

**Keywords:** Pandemic (H1N1) 2009, H1N1, hemagglutinin, receptor specificity, infectivity and transmission in avian species, viruses, influenza, expedited, dispatch

## Abstract

We demonstrate that the novel pandemic influenza (H1N1) viruses have human virus–like receptor specificity and can no longer replicate in aquatic waterfowl, their historic natural reservoir. The biological properties of these viruses are consistent with those of their phylogenetic progenitors, indicating longstanding adaptation to mammals.

In 2009, a new H1N1 influenza virus (pandemic [H1N1] 2009) emerged in Mexico, spread to the United States (*1*), and subsequently caused the first influenza pandemic of the 21st century (*2*). The emergence of pandemic (H1N1) 2009 virus is imperfectly understood, but an early switch in hemagglutinin (HA) receptor specificity is essential to allow interspecies transmission (*3*–*5*).

Pandemic (H1N1) 2009 virus strains were recently reported to be reassortants of the North American and European swine lineages (*6*). Phylogenetic evidence suggests that this reassortment event occurred 10–17 years ago (*7*). These data suggest that the current pandemic (H1N1) 2009 virus strains should have receptor specificity typically found in the HA of mammalian viruses (Neu5Acα2,6Gal). In addition, they may have lost the ability to replicate in avian hosts, the natural reservoir species. To test these hypotheses, we examined the biological properties of pandemic (H1N1) 2009 virus, including receptor specificity, erythrocyte binding, and ability to replicate in avian species.

## The Study

We first tested species-specific erythrocyte agglutination by the pandemic (H1N1) 2009 isolates A/California/04/2009 and A/Tennessee/1-560/2009 and by other isolates from humans, swine, and birds (Table 1). The pandemic (H1N1) 2009 isolates showed reduced or absent agglutination of goose and chicken erythrocytes. Human and swine H1N1 viruses were agglutinated by turkey, guinea pig, chicken, and goose erythrocytes, and all erythrocytes we tested except those of swine were agglutinated by avian isolates (Table 1).

**Table 1:** Erythrocyte agglutination by representative human, pandemic, swine, and avian H1 influenza virus isolates

Virus isolate	Subtype	Hemagglutination titer of erythrocytes from indicated species, HAU*†					
		Turkey‡	Guinea pig‡	Chicken§	Goose§	Horse¶	Swine#
Human isolates							
A/Brisbane/59/2007	H1N1	64	64	64	64	<2	<2
A/New Jersey/15/2007	H1N1	32	32	16	16	<2	<2
Pandemic isolates							
A/California/04/2009	H1N1	64	64	4	16	<2	<2
A/Tennessee/1-560/2009	H1N1	32	32	<2	8	<2	<2
Swine isolates							
A/swine/North Carolina/007270/2008	H1N1	32	64	8	16	<2	<2
A/swine/Iowa/003479/2009	H1N1	64	64	32	32	2	<2
Avian isolates							
A/mallard/Alberta/66/2007	H1N4	64	64	32	32	16	<2
A/mallard/Alberta/496/2008	H1N4	64	64	32	32	16	<2

*HAU, hemagglutination units. †Titers are expressed as the reciprocal of the highest virus dilution that yields complete HA agglutination. ‡Neu5Acα2,6Gal > Neu5Acα2,3Gal. §Neu5Acα2,6Gal < Neu5Acα2,3Gal. ¶Neu5Gc2,3Gal. #Neu5Gcα2,6Gal > Neu5Gcα2,3Gal.

We next measured the receptor binding of the 2 pandemic (H1N1) 2009 isolates to sialic substrates, both natural (fetuin) and synthetic (3′-sialyllactose [3′SL] and 6′-sialyllactosamine [6′SLN] attached to a polyacrylic carrier) (Figure). The binding pattern to fetuin was identical among all isolates tested (association constant *K_ass_* ≈ 5.8 ± 0.5, 1/μM sialic acid). The currently circulating human and pandemic influenza (H1N1) viruses showed a preference for 6′SLN and negligible binding to the avian-type 3′SL. A similar pattern was observed for 2 recent swine viruses, which bound only to 6′SLN receptors with nearly equal affinity as pandemic (H1N1) 2009 isolates. As expected, the 2 avian H1 viruses bound strongly only to 3′SL (Figure).

**Figure:**
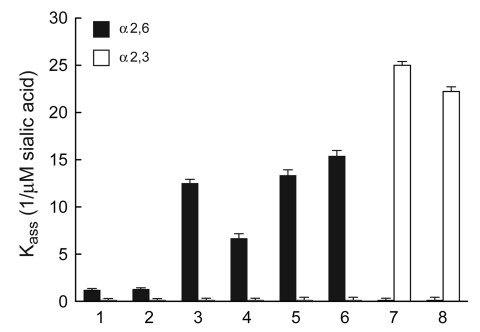
Receptor specificity of human, pandemic, swine, and avian H1 influenza viruses. Association constants (*K_ass_*, 1/μM sialic acid) of virus complexes with sialylglycopolymers conjugated to 3′-sialyllactose (avian-like Neu5Acα2,3Gal-containing receptor, white bars) and 6′-sialyllactosamine (human-like Neu5Acα2,6Gal-containing receptor, black bars). Higher *K_ass_* values indicate stronger binding. Values are the mean ± SD of 4 independent experiments (1/μM sialic acid). 1, A/Brisbane/59/2007; 2, A/New Jersey/15/2007; 3, A/California/04/2009; 4, A/Tennessee/1-560/2009; 5, A/swine/North Carolina/007270/2008; 6, A/swine/Iowa/003479/2009; 7, A/mallard/Alberta/66/2007; 8, A/mallard/Alberta/496/2008.

To assess the infectivity and pathogenicity of pandemic (H1N1) 2009 virus strain A/California/04/2009 in terrestrial (chickens, quails) and aquatic (domestic and wild ducks) avian species, we inoculated 10 birds of each species by intranasal, intraocular, and intratracheal instillation with ≈10^6.0^ of the 50% egg infectious dose (EID_50_) of the virus. We then observed the birds for the next 2 weeks for death and for viral shedding and signs of illness. No birds showed obvious clinical signs of disease. Virus was detected only on postinoculation day 1 in infected chickens and ducks and only in tracheal samples at low titers (<1.7 log_10_ of the EID_50_/mL [*8*]) (Table 2). However, no later shedding of virus was observed, indicating that the virus detected on postinoculation day 1 could have been caused by residual virus particles after inoculation. In contrast, our results revealed that the A/California/04/2009 strain efficiently infected quails with significantly higher titers (<3.4 log_10_ EID_50_/mL until postinoculation day 5; p<0.05) in both oropharyngeal and cloacal swab specimens (Table 2). The virus was detected in the trachea (1.7 log_10_ EID_50_/g), lungs (2.3 log_10_ EID_50_/g), and cecal tonsil (0.8 log_10_ EID_50_/g) of quails on postinoculation day 5.

**Table 2:** Replication and transmission of influenza virus A/California/04/2009 (H1N1) in various bird species

Common name (genus and species)	Virus titer*								Transmission†
	1 dpi			3 dpi			5 dpi		
	Oropharynx	Cloaca		Oropharynx	Cloaca		Oropharynx	Cloaca	
Chicken (*Gallus gallus domesticus*)	1.7 ± 0.5	<		<	<		<	<	0
Domestic duck (*Anas platyrhynchos*)	1.7 ± 0.5	<		<	<		<	<	0
Wild duck (*Anas platyrhynchos*)	1.3 ± 0.0	<		<	<		<	<	0
Quail (*Coturnix japonica*)	3.4 ± 0.9‡	<		2.0 ± 1.3‡	0.8 ± 0.9		1.3 ± 0.9	1.3 ± 0.9	33‡

*dpi, days postinoculation; <, titer below limit of detection (<0.75 log_10_EID_50_/mL). Virus titers were determined in eggs and are expressed as the log_10_ 50% egg infectious dose (EID_50_)/mL (*8*). Data are presented as means ± standard deviation of titers of positive samples (≥0.75 log_10_ EID_50_/mL). †Percentage of contact birds from which virus was isolated. ‡p<0.05, by 1-way analysis of variance.

The potential bird-to-bird intraspecies transmission of the A/California/04/2009 pandemic (H1N1) 2009 virus strain in avian species was also examined by introducing 3 contact birds to the inoculated birds’ cages on postinoculation day 1. There was no subsequent evidence of viral shedding through the upper respiratory tract or fecal-oral route in any group of birds except 1 of 3 contact quails (Table 2). Oropharyngeal virus titers in this quail were l.7 and 1.5 log_10_ EID_50_/mL on postinoculation days 3 and 5, indicating that productive viral replication was occurring.

## Conclusions

The A/California/04/2009 pandemic (H1N1) 2009 virus strain showed minimal replication and no transmission in chickens and ducks (domestic and wild), but the virus replicated and had limited transmissibility in quails. Our finding is consistent with those of Swayne et al. (*9*). The inability of the virus to replicate efficiently in chickens and ducks could very well be linked to its human virus–like receptor recognition.

The ability of influenza A viruses to agglutinate erythrocytes from a variety of hosts may reflect the viruses’ receptor specificity (*10*,*11*). We observed similar binding patterns for the mammalian influenza (H1N1) viruses, with the exception that the pandemic strains had reduced binding to chicken erythrocytes. This binding pattern was also observed with 1 of the swine isolates, suggesting it might be a trait of swine-adapted viruses. Taken together, a difference in the hemadsorption phenotype observed with erythrocytes from species with either less Neu5Acα2,6Gal or less Neu5Ac linkage overall could be explained by the mammalian origin of the novel pandemic (H1N1) 2009 influenza viruses.

To test this possibility, we measured the HA affinity of H1 influenza viruses from various species of origin for synthetic receptor analogues. All mammalian H1 viruses showed a typical human virus–like preference for the Neu5Acα2,6Gal-containing receptor 6′SLN. Compared with the currently circulating H1N1 human viruses, both pandemic (H1N1) 2009 strains and contemporary swine influenza virus (H1N1) strains were able to bind substantially more strongly (5–12×) to an α2,6-containing glycopolymer; the currently circulating subtype H1N1 human viruses are strictly adapted to this receptor (*12*). This feature demonstrated that pandemic H1N1 strains, which have a HA gene of swine lineage, have retained their current swine virus–like binding characteristics despite their efficient spread in humans.

To identify substitutions in the HA molecule that could be responsible for the human-like receptor binding phenotype of the pandemic and contemporary swine influenza (H1N1) isolates, we compared the H1 HA sequences deposited in the Influenza Research Database (www.fludb.org). We observed that 99.99% of all swine viruses isolated after 1980 have Asp190 or Asn190. HA sequences of swine viruses isolated before 2000 harbor Gly225, whereas 92.3% of more recent classical swine viruses have Asp225. Crystallographic analysis of human and swine H1 HA has shown that Asp225 and Asp190 are responsible for human virus–like receptor specificity (*13*). Therefore, the human-like amino acids encoded at HA positions 190 and 225 in the novel pandemic and swine influenza (H1N1) viruses may at least partially explain their innate affinity for the human-type receptor.

Recent phylogenetic analysis showed that each segment of the pandemic (H1N1) 2009 virus is nested within a well-established swine influenza lineage for >10 years before the recent outbreak (*7*). Hence, the ancestors of this virus circulated undetected for about a decade before the virus emerged in humans. Our finding that contemporary swine viruses acquired the ability to recognize 6′SLN with at least 5-fold higher affinity than did human strains and completely lost the ability to bind to Neu5Acα2,3Gal provides clear evidence to support this hypothesis. It is possible that the progenitors of pandemic (H1N1) 2009 virus were accumulating enough mammal-associated changes to allow a refinement of their receptor-binding properties. Our findings substantiate that strong mammalian-like receptor specificity is a critical barrier to infection of various hosts with pandemic (H1N1) 2009 virus. Other biological factors associated with their adaptation and tissue tropism in humans will likely be identified in the future.
